# Effectively Addressing Human Immunodeficiency Virus Disparities Affecting US Black Women

**DOI:** 10.1089/heq.2018.0038

**Published:** 2018-11-16

**Authors:** Erin L.P. Bradley, Angelica Geter, Ashley C. Lima, Madeline Y. Sutton, Donna Hubbard McCree

**Affiliations:** Division of HIV/AIDS Prevention, National Centers for HIV, Viral Hepatitis, STD, and TB Prevention, Centers for Disease Control and Prevention, Atlanta, Georgia.

**Keywords:** black/African American women, health disparity, health equity, HIV/AIDS, social determinants, United States

## Abstract

Black women have disproportionately higher rates of human immunodeficiency virus (HIV) infection, and low percentages being linked to care and becoming virally suppressed, compared with women of other races/ethnicities. To date, few evidence-based HIV prevention and care interventions tailored for black women exist. We highlight three essential factors to consider in designing culturally and gender-appropriate studies to address HIV-related disparities affecting black women: (1) social determinants of HIV risk, (2) determinants of equity, and (3) perceptions of black women's sexuality. Synergy between a strong evidence base and developing strong partnerships could accelerate progress toward HIV-related health equity for black women.

## Introduction

Disparity, which can be defined as a lack of similarity or equality; inequality; difference,^1^ is a word that has characterized human immunodeficiency virus (HIV) diagnoses and outcomes for black/African American women (referred to as black women) since the 1980s, when acquired immunodeficiency syndrome (AIDS) was first recognized in the United States.^2^ Although black women experienced the largest decline in HIV diagnoses rates among women from 2010 to 2014, annual disparities between black women and their white counterparts persist.^3^ In 2016, 61% of HIV diagnoses in women occurred among black women, although they comprised 13% of the US female population.^4^ The diagnosis rate among black women in 2016 was 15 times that seen in white women.^4^ Recent estimates show 1 in 54 black women compared with 1 in 941 white women may be diagnosed with HIV in their lifetime, if rates remain the same.^5^

The causes of these disparities are complex, extend beyond individual risk behaviors (e.g., condom use), and include social and structural factors, such as inequitable access to health care, stigma, and higher community levels of some sexually transmitted infections (STIs) that increase HIV risk.^6,7^ Compared with women in other racial/ethnic groups, black women report higher levels of condom use during heterosexual intercourse,^6,8,9^ the primary mode of HIV transmission among women.^4^ This underscores that the socioecological environments in which risk behaviors occur may increase black women's risk for HIV infection.^6^

Benefits of early diagnosis, linkage to care, viral suppression, and pre-exposure prophylaxis (PrEP, a medication to prevent HIV infection) in reducing HIV transmission are well known.^10^ However, PrEP use among black women is virtually nonexistent,^11^ possibly due to a lack of targeted efforts for this group. Moreover, results from research studies and national surveillance data highlight key social and structural challenges hindering care seeking among black women living with HIV.^12^ Compared with their white and Hispanic/Latina counterparts, a lower percentage of black women are linked to HIV care^13,14^ and virally suppressed.^13,15^ These disparities warrant concerted efforts, including prioritizing HIV prevention, treatment, and care, to reduce the disproportionate HIV burden for black women.

Effectively addressing HIV-related disparities will depend, in part, on developing and implementing tailored approaches for black women that are informed by a strong evidence base. To date, there are few culturally tailored, evidence-based HIV interventions for black women. In this perspectives article, we highlight three essential factors for researchers to consider in designing culturally and gender-appropriate studies to address HIV-related disparities affecting black women: (1) social determinants of HIV risk, (2) determinants of equity for black women, and (3) perceptions of black women's sexuality.

## Social Determinants of HIV

The existing body of research suggests social determi-nants of HIV (SDH) for black women include proximate and distal factors that contribute to disparities between black and white women. For example, higher incarceration and mortality rates among black men contribute to a sex ratio imbalance that may inadvertently promote concurrent partnerships.^6,16^ Assortative mixing patterns (having partners of the same race) in networks with a higher prevalence of undiagnosed and untreated HIV/STIs, especially in the southern United States, increase transmission risk, even with less risky sexual behavior.^17,18^ The effects of these partnership and network-related factors are exacerbated by poverty.^6,19^ Other factors such as HIV stigma,^20^ difficulty accessing quality health care, or navigating health care systems^12^ can worsen HIV prevention and care engagement outcomes for black women.

It is imperative that knowledge from SDH research be reflected in all aspects of research studies with black women, including developing research questions, designing and implementing studies, and interpreting findings. Just as researchers and practitioners have aligned HIV prevention and care efforts with current scientific knowledge (e.g., prioritizing sustained viral suppression, PrEP use, and integrated behavioral and biomedical approaches), disparities-focused research and programmatic efforts must also align with the current state of the science regarding social and structural factors that best explain disparate rates of HIV diagnoses and poor outcomes for black women (Table 1).

**Table 1.** **Examples of Culturally and Gender-Appropriate Research to Advance HIV-Related Health Equity for Black Women**

Disparities-focused study aim	Study that addresses key social and structural factors	Collaborative and multidisciplinary approach
**HIV care engagement**		
Improve black women's health care engagement by **equipping providers and frontline staff** with knowledge, attitudes, and practices to provide culturally and gender-appropriate^a^ care Key factors: • Determinants of equity • Perceptions of black women's sexuality	1. Develop and test the efficacy of culturally and gender-appropriate patient care education/training for providers and frontline staff *Note:* Formative research with HIV-positive and HIV-negative black women (engaged in care and not engaged) that identifies barriers, facilitators (including resilience), and appropriate solutions is likely needed to inform the development of training materials 2. Implement and evaluate the effect of requirements to complete CME credits in areas related to racial or cultural bias on black women's care engagement and perceptions of the quality of care received 3. Assess attitudes and beliefs of providers and frontline staff regarding black women's sexuality, and ways they might affect interactions with and recommendations or treatment plans for black women *Note*: Study design could be informed by experimental social psychology literature on prejudice or implicit bias	Key partners for PIs to involve might include: • Black women (HIV-positive and/or HIV-negative) and advocates—*inform appropriateness of research questions, methods, interpretation of findings, implications, solutions* • Trusted community partners such as women-serving, faith-based, or other organizations actively engaged with black women—*engaging black women in the research process* • Behavioral scientist, health education specialist, health communications specialist—*development and testing of training* • Health care provider—*clinical practice perspective* • Medical sociologist or other social scientist (e.g., social psychologist, anthropologist, historian)—*knowledge of historical context affecting black women's engagement with health care providers and systems*
Improve black women's health care engagement by **reducing barriers** to accessing high-quality HIV-related prevention and care services Key factors: • Social determinants of health • Determinants of equity	1. Conduct an organization-level evaluation of health care facility policies and practices to identify aspects that might impede care access for some black women (e.g., hours of operation, efficiency of operations [affordable service, but lengthy wait times], costs and/or difficulty navigating payment assistance processes, negative patient–staff interactions)	Key partners for PIs to involve might include: • Black women (HIV-positive and/or HIV-negative) and advocates—*insight regarding barriers and facilitators* • Trusted community partners—*engaging black women in the research process* • Medical sociologist or other social scientist—*knowledge of historical context affecting black women's engagement with health care providers and systems* • Health care provider—*clinical practice perspective* • Health policy analyst—*expertise in evaluating organizational policies*
**HIV testing and linkage to care or prevention services**		
**Reduce HIV/STIs in black sexualnetworks** in geographic areas with high HIV prevalence or incidence Key factor: • Social determinants of health	1. Evaluate community-based (including grassroots) “test and treat” strategies that identify undiagnosed persons, link HIV-positive persons to HIV care and high-risk negative persons to prevention services (e.g., PrEP).	Key partners for PIs to involve might include: • Black women (HIV positive and/or HIV negative) and advocates—*insight regarding strategies* • Health departments, other providers, CBOs—*testing and care* • Trusted community partners—*engaging community members*
**Improve access** to PrEP for black women Key factor: • Social determinants of HIV	1. Assess the economic cost and benefit/public health impact of scaling up PrEP for women in high-prevalence areas 2. Model the effects of scaling up PrEP for black women on HIV incidence and on disparities 3. Evaluate preparedness of women's health care providers regarding PrEP readiness screening and how to prescribe/help women navigate PrEP access	Key partners for PIs to involve might include: • Health economist—*expertise in cost analysis* • Clinical care providers, including physicians, nurse practitioners and physician assistants—*operationalizing increased PrEP access opportunities for black women*

Funding for culturally and gender-appropriate research with black women will require grassroots efforts by black women in local communities and in gatekeeper positions to raise awareness and engage policy makers to request funding.

^a^ Culturally and gender-appropriate care considers intersectionality (combined effects of race and gender) that can create experiences for black women that differ from those of men or other women. For example, conscious or unconscious bias informed by hypersexualized images or other negative perceptions of black persons might influence provider engagement and treatment plans for black persons. For black women, in addition to race, gender-specific attitudes or beliefs might influence provider engagement and treatment plans.

CBO, community based organization; CME, continuing medical education; HIV, human immunodeficiency virus; PI, principal investigator; PrEP, pre-exposure prophylaxis; STI, sexually transmitted infection.

## Determinants of Equity

Devoting research attention to determinants of equity that are linked to SDH is also fundamental. While SDH are contextual factors that explain why black women are more likely than white women to acquire HIV even with less risky behavior, social determinants of equity explain *why contexts differ* for many black women compared with their white counterparts.^21^ For example, access to high-quality HIV prevention and care services (structural factor) can differ for some black women compared with white women because of racism (determinant of equity). Racism, defined by Jones as a system that “structures opportunity and assigns value based on the social interpretation of how one looks,”^21^ is a determinant of equity with historical foundations for black women that date back to slavery and continue to impact modern-day health care and sexual health.^21,22^ Gynecologic surgeries performed on enslaved black women without consent or anesthesia, and forced permanent sterilizations mostly on black and Hispanic women/Latinas to decrease “undesirable” minority children from “promiscuous” women,^22^ provide some historical context for understanding medical distrust reported by some black women.^23^

Additionally, racism in health care settings (explicit or implicit) can discourage black women from initiating or continuing care, and can negatively affect the quality of their care.^23^ Socioecological models can provide frameworks for understanding and intervening on racism and key SDH at individual, interpersonal, community, and societal levels.^24^ Collaborative, multidisciplinary, and systems-level research that ethically engages black women and their health care providers to further explore racism and distrust, and remove them as health care barriers, is vitally needed to bolster health care engagement (Table 1).

## Perceptions of Black Women's Sexuality

Many researchers, practitioners, and policy makers may be unaware of ways that historical representations of black women's sexuality can shape perceptions of HIV risk. Historically, black women's sexuality has not been their own to define. Stripped of their sexual agency during slavery, some of black women's earliest experiences in the United States included public exhibition as sexual curiosities,^25^ sexual abuse and reproductive exploitation,^26^ and involuntary scientific experimentation.^22^ The residual effects of black women's sexual exploitation range from advances in modern-day reproductive medicine^22^ to ubiquitous misperceptions and stereotypes that overemphasize black women's sexuality.^25^

Dismantling faulty perceptions about black women's sexuality is key because it perpetuates a view of black women as more “promiscuous,” “irresponsible,” or “reckless” than women of other races/ethnicities, despite research findings that demonstrate otherwise.^6,8,9^ These stereotypes have the potential to bias the research agenda away from addressing SDH to a disproportionate focus on individual behavior. Similarly, these stereotypes create the potential for misperceptions to influence practitioners' assumptions about, interactions with, and recommendations or treatment plans for black women, even outside of the practitioners' awareness or intent.^23^ Consequently, engaging sociologists, anthropologists, and historians in multidisciplinary research can yield valuable insight for practitioners and policy makers who are informed by research findings (Table 1).

## Conclusion

Reducing or eliminating the disproportionate adverse effects of HIV-related disparities on black women is achievable, and could be considered a public health imperative and an ethical responsibility. Achieving equity will require an intentional paradigm shift in the current approach to HIV prevention and care research with black women at risk of or living with HIV. This means designing studies that demonstrate careful consideration of gender and cultural factors, and prioritize addressing social and structural factors that increase black women's vulnerability to HIV infection or poor outcomes (Table 1). Attention to key SDH, racism as a determinant of equity, and perceptions of black women's sexuality is important. Using social ecological frameworks provides a comprehensive lens for developing research questions, designing and implementing studies, and interpreting findings.^24^ Furthermore, conducting research to identify where disparities are most pronounced (e.g., specific subpopulations or geographic locations) and monitoring progress in closing gaps over time are paramount.^3^

Research highlighting the critical role of sexual network characteristics,^6,16–19^ social and economic factors that affect sexual partnerships,^6,19^ and challenges accessing or utilizing quality health care^12^ has laid the foundation for disparities-focused epidemiologic and intervention research with black women. However, additional studies that evaluate strategies to address SDH are needed. For example, microenterprise approaches (e.g., business education and support, financial counseling, loans) to address poverty as a key SDH have shown promise in studies internationally and warrant additional research attention domestically.^27^ Health services research to increase PrEP uptake among HIV-negative black women and improve care linkage and retention for black women living with HIV also provides important opportunities to address disparities. Valuable insight may also be gained from identifying factors that promote resilience among black women (e.g., spirituality, social support). Therefore, opportunities to build upon promising findings and strengthen the evidence base to decrease HIV gaps for black women remain.

Benefits of advances in HIV prevention and care are not being experienced equally across populations.^4^ Adjustments are needed to ensure that black women are not left behind in global efforts to eliminate new HIV infections, AIDS-related deaths, and HIV/AIDS-related discrimination. Promoting an evidence-informed narrative about black women's HIV-related risk and aligning research priorities with current scientific evidence are essential, and will provide a strong foundation for creating culturally tailored interventions to eliminate disparities. Additionally, a collective investment in developing and implementing a suite of culturally and gender-appropriate public health interventions is needed. Synergy between a strong evidence base and strong partnerships between researchers, clinicians, public health agencies, professional societies, community members and advocates, and entities from public and private sectors could accelerate progress toward effectively addressing social and structural factors that create or sustain HIV-related disparities that negatively affect black women.

## Abbreviations Used

AIDS: acquired immunodeficiency syndrome
CME: continuing medical education
HIV: human immunodeficiency virus
PrEP: pre-exposure prophylaxis
SDH: social determinants of HIV
STI: sexually transmitted infection
